# Formononetin: A Review of Its Anticancer Potentials and Mechanisms

**DOI:** 10.3389/fphar.2019.00820

**Published:** 2019-07-26

**Authors:** Kai-Ching Tay, Loh Teng-Hern Tan, Chim Kei Chan, Sok Lai Hong, Kok-Gan Chan, Wei Hsum Yap, Priyia Pusparajah, Learn-Han Lee, Bey-Hing Goh

**Affiliations:** ^1^Biofunctional Molecule Exploratory (BMEX) Research Group, School of Pharmacy, Monash University Malaysia, Bandar Sunway, Malaysia; ^2^Novel Bacteria and Drug Discovery (NBDD) Research Group, Microbiome and Bioresource Research Strength Jeffrey Cheah School of Medicine and Health Sciences, Monash University Malaysia, Bandar Sunway, Malaysia; ^3^Institute of Biomedical and Pharmaceutical Sciences, Guangdong University of Technology, Guangzhou, China; ^4^de Duve Institute, Brussels, Belgium; ^5^Centre for Research Services, Institute of Research Management and Services, University of Malaya, Kuala Lumpur, Malaysia; ^6^Division of Genetics and Molecular Biology, Institute of Biological Sciences, Faculty of Science, University of Malaya, Kuala Lumpur, Malaysia; ^7^International Genome Centre, Jiangsu University, Zhenjiang, China; ^8^School of Biosciences, Taylor’s University, Subang Jaya, Malaysia; ^9^Medical Health and Translational Research Group (MHTR), Jeffrey Cheah School of Medicine and Health Sciences, Monash University Malaysia, Bandar Sunway, Malaysia; ^10^Institute of Pharmaceutical Science, University of Veterinary and Animal Science, Lahore, Pakistan

**Keywords:** formononetin, anticancer, antitumor, apoptosis, anti-metastasis

## Abstract

Cancer, a complex yet common disease, is caused by uncontrolled cell division and abnormal cell growth due to a variety of gene mutations. Seeking effective treatments for cancer is a major research focus, as the incidence of cancer is on the rise and drug resistance to existing anti-cancer drugs is major concern. Natural products have the potential to yield unique molecules and combinations of substances that may be effective against cancer with relatively low toxicity/better side effect profile compared to standard anticancer therapy. Drug discovery work with natural products has demonstrated that natural compounds display a wide range of biological activities correlating to anticancer effects. In this review, we discuss formononetin (C_16_H_12_O_4_), which originates mainly from red clovers and the Chinese herb *Astragalus membranaceus*. The compound comes from a class of 7-hydroisoflavones with a substitution of methoxy group at position 4. Formononetin elicits antitumorigenic properties *in vitro* and *in vivo* by modulating numerous signaling pathways to induce cell apoptosis (by intrinsic pathway involving Bax, Bcl-2, and caspase-3 proteins) and cell cycle arrest (by regulating mediators like cyclin A, cyclin B1, and cyclin D1), suppress cell proliferation [by signal transducer and activator of transcription (STAT) activation, phosphatidylinositol 3-kinase/protein kinase-B (PI3K/AKT), and mitogen-activated protein kinase (MAPK) signaling pathway], and inhibit cell invasion [by regulating growth factors vascular endothelial growth factor (VEGF) and Fibroblast growth factor 2 (FGF2), and matrix metalloproteinase (MMP)-2 and MMP-9 proteins]. Co-treatment with other chemotherapy drugs such as bortezomib, LY2940002, U0126, sunitinib, epirubicin, doxorubicin, temozolomide, and metformin enhances the anticancer potential of both formononetin and the respective drugs through synergistic effect. Compiling the evidence thus far highlights the potential of formononetin to be a promising candidate for chemoprevention and chemotherapy.

## Introduction

Cancer is one of the leading causes of death worldwide, with the current rate of developing cancer listed at 1 in 5 for men and 1 in 6 for women, while cancer mortality is currently 1 in 8 among men and 1 in 10 among women. It was estimated to cause 9.6 million deaths in 2018 and that approximately 18.1 million new cancer cases would occur in 2018 (Bray et al., 2018). The most common cancers include lung cancer (11.6% of total new cases), breast cancer (11.6% of total new cases), prostate cancer (7.1% of total new cases), colorectal cancer (6.1% of total new cases), stomach cancer (5.7% of total new cases), and liver cancer (4.7% of total new cases) in both sexes combined (Bray et al., 2018). Despite the modern technology and multimodal treatments available, intensive research and development of alternative effective anticancer drugs is still ongoing to find a cure for this disease. Natural products are recognized as an indispensable source for discovery and development of chemopreventive and chemotherapeutic agents (Goh et al., 2019; Tan et al., 2017; Tan et al.,2019). Currently, around 75% of the clinically used anticancer drugs are derived from natural bioresources, including plants, animals, and microorganisms (Newman and Cragg, 2016). Ever since ancient times, people have used plants for various medicinal uses, including treatment for injuries, ailments, and general health well-being (Tan et al., 2015). The first records of plants used as traditional medicines, written in cuneiform, dates back to 2600 B.C. in Mesopotamia (Cragg and Newman, 2005a). Plants represent a great source of biologically active natural products (Chan et al., 2016; Ma et al., 2018; Tang et al., 2016), and many of these plant-derived natural products and derivatives have been developed into what is now the standard repertoire of cancer chemotherapy available today, such as paclitaxel, vinblastine, and etoposide (Cragg and Newman, 2005b).

*Astragalus membranaceus* (Huangqi) has been used widely and commonly in China as a traditional herb for many centuries and is believed to boost the immune system; scientific analysis has revealed that this species contains a plethora of flavonoids, where more than 200 compounds were identified (Liu et al., 2017). Formononetin is one of the flavonoids identified in *A. membranaceus* and has recently gained attention for its antitumor and neuroprotective properties (Chen et al., 2013; El-Bakoush and Olajide, 2018; Li et al., 2018). Many recent studies showed that formononetin possesses great potential in blocking proliferation, such as by inducing apoptosis of tumor cells *via* various signaling pathways (Kim et al., 2018a; Park et al., 2018; Zhang et al., 2018a). Formononetin exhibits cytotoxicity towards various cancer cells, including nasopharyngeal carcinoma cells, and multiple myeloma cells, showing that formononetin could be an attractive drug candidate for cancer therapy (Qi et al., 2016; Kim et al., 2018a). In this review, we summarize the anti-cancer properties of formononetin and its underlying mechanisms reported based on *in vitro* and *in vivo* experimental evidences. In turn, the compilation of these scientific evidences of formononetin in anticancer properties could facilitate future research to further explore potential therapeutic targets of formononetin in cancer therapy.

## Overview of Formononetin

**Figure 1:**
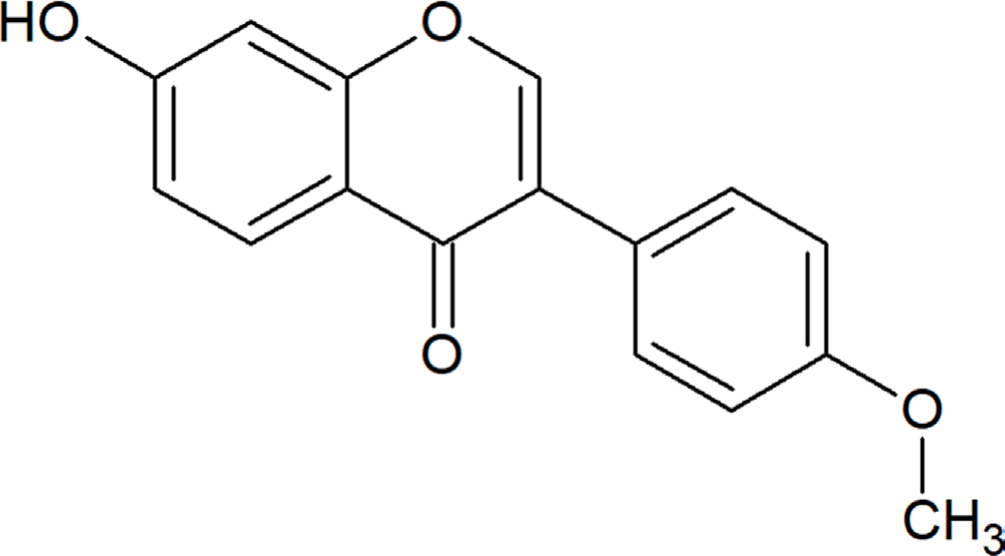
Chemical structure of formononetin.

Formononetin [IUPAC: 7-hydroxy-3-(4-methoxyphenyl) chromen-4-one], with a molecular weight of 268.268 g/mol, is an *O*-methylated isoflavone that is widely present in legumes, many species of clovers especially red clovers *Trifolium pratense* L., and the traditional Chinese herb *Astragalus membranaceus* (Fisch). Bunge (Heinonen et al., 2004; Zhang et al., 2018a). **Figure 1** depicts the chemical structure of formononetin. **Table 1** tabulates a list of reported sources of formononetin. In the leguminous plant, formononetin is an important intermediate for the biosynthesis of phytoalexins, which function to defend the plant from stressful environments or diseases. In leguminous plants, formononetin is mainly synthesized from 2,7,4-trihydroxy-isoflavone by enzyme 2,7,4’-trihydroxyisoflavanone 4’-*O*-methyltansferases (HI_4_’OMTs). Meanwhile, in *Pueraria lobata*, formononetin is synthesized from daidzein by *Pueraria lobata* *O*-methyltransferases (PlOMT9) (Akashi et al., 2000; Li et al., 2016a).

**Table 1:** The different isolation sources of formononetin.

Plant species	Part of plant isolated	References
*Actaea racemosa* L. (black cohosh)	Roots, rhizomes	(Jiang et al., 2006)
*Amorpha fruticosa* L.	Roots, leaves	(Kim et al., 2011; Cui et al., 2017)
*Andira inermis* (Wright) DC.	Stems, leaves	(Kraft et al., 2000)
*Astragalus membranaceus* (Fisch). Bunge	Roots	(Lee et al., 2017)
*Astragalus mongholicus* Bunge	Roots	(Yu et al., 2005)
*Baptisia australis* (L). R. Br.	Stem, leaves, flower	(Lebreton et al., 1967)
*Bolusanthus speciosus* (Bolus) Harms	Root wood	(Erasto et al., 2004)
*Cicer arietinum* L. (Chickpea)	Germinated seeds, stem	(Ingham, 1976; Milán-Noris et al., 2018)
*Dalbergia cearensis* Ducke	Wood	(Salignac De Souza et al., 1975)
*Dalbergia ecastophyllum* (L). Taub	Wood	(De Abreu Matos et al., 1975)
*Dalbergia stevensonii* Standl.	Bark, heartwood	(Donnelly et al., 1973)
*Euphorbia portlandica* L.	air-dried whole plant	(Madureira et al., 2004)
*Glycyrrhiza glabra* L.	Roots	(Benedec et al., 2012; Fang et al., 2016)
*Glycyrrhiza pallidiflora* Maxim.	Roots	(Li et al., 2002; Shults et al., 2017)
*Glycyrrhiza uralensis *Fisch.	Roots	(Fang et al., 2016)
*Medicago sativa* L. (Alfalfa)	Root exudate, aerial parts	(Dakora et al., 1993; Stochmal et al., 2001)
*Ononis spinosa* L.	Roots	(Benedec et al., 2012)
*Pterocarpus indicus* Willd.	Heartwood	(Cooke and Rae, 1964)
*Pueraria lobata* (Willd). Ohwi	Roots, vines	(Wagle et al., 2019)
*Sophora flavescens* Aiton	Roots	(Huang et al., 2017; Kim et al., 2018b)
*Trifolium pratense* L.	Aerial parts of plant	(Spagnuolo et al., 2014; Kolodziejczyk-Czepas et al., 2018)
*Trifolium subterraneum* L.	Leaves	(Tava et al., 2016)
*Virola caducifolia* W. A. Rodrigues	Trunk wood	(Braz Filho et al., 1977)
*Virola multinervia* Ducke	Wood	(Braz Filho et al., 1977)

The formononetin concentration in red clover ranges from 3.4 to 6.8 mg/g dry mass (Mcmurray et al., 1986). Given the low yield from extraction and limited amount present in raw plant materials, chemical synthesis of formononetin has also been the subject of research due to its clinically important biological activities (Chang et al., 1994; Li et al., 2009). In 1994, the synthesis of formononetin was greatly improved in terms of time and yield by the use of a conventional microwave synthesis method. Formononetin was chemically synthesized using low-cost materials to achieve a higher yield (overall 40%). This rapid and cost-effective synthesis method may facilitate more preclinical investigations of the diverse pharmacological properties of formononetin (Chang et al., 1994). To date, there are several patents filed on the synthesis methods of formononetin with advantages of simple, low cost, high yield, and purity (Fu et al., 2011; Guo et al., 2011).

Given that the structure of formononetin is relatively similar to endogenous oestrogen (estradiol), formononetin is known to be one of the phytoestrogens, which is able to bind to oestrogen receptors, namely, estrogen receptors α and β. Due to its phytoestrogenic properties and diverse biological activities, formononetin has gained the attention of researchers from the field of natural products, especially those working on anticancer drug discovery. This is because an early association has been observed between the epidemiological evidence of lower breast cancer incidence within the Asian population who consume higher dietary concentration of soy products containing high phytoestrogens content as compared to the Western population (Watanabe et al., 2002; Korde et al., 2009).In contrast to the beneficial health claims, phytoestrogens have also been linked with potential adverse effects, particularly its role in breast cancer remains controversial, since the oestrogenic properties of isoflavones may increase the risk of tumor recurrence (Messina et al., 2006). In spite of some fears, there is no solid evidence indicating that a diet rich in isoflavones increases risk of breast cancer. In fact, numerous studies have reported the anticancer properties of formononetin in both *in vitro* and *in vivo* experiments. Moreover, formononetin is predominantly metabolized by cytochrome P450 enzymes upon consumption into daidzein, and daidzein is further metabolized into equol (Dickinson et al., 1988; Tolleson et al., 2002). These estrogenic metabolites also possess anticancer properties, such as inducing cell apoptosis (Jin et al., 2010; Zongliang et al., 2016). In this regard, several studies also designed and synthesized a series of novel formononetin derivatives or analogues exerting interesting anticancer properties (Yang et al., 2008; Ren et al., 2012; Fu et al., 2017; Lin et al., 2017).

## Anticancer Effects of Formononetin

### *In Vitro* Studies

Formononetin has been shown to exhibit anticancer effect on various cancer cells, including colon, breast, prostate, breast, nasopharyngeal, and lung cancer cells. **Table 2** tabulates the *in vitro* studies on the dosage, efficacy, and potential molecular mechanisms of formononetin on different cancer cells. In different cancer cells, differential anticancer effects were observed on exposure to formononetin with the majority of the studies testing formononetin at 1–200 µM (0.3–53.7 µg/ml). As shown in **Table 2**, formononetin exhibits IC_50_ between the range of 10–300 μM, showcasing the potency of formononetin in inhibiting various cancer cells. In addition, formononetin exhibits a great variety of anticancer action on different cancer cells. It has been suggested that formononetin could regulate various molecular signaling pathways including the proliferation, cell cycle regulation, apoptosis, angiogenesis, and metastasis of cancer cells.

**Table 2:** The cytotoxic effects of formononetin against cancer cells in *in vitro* experiments*.*

Cell lines		Mechanisms of action	Concentrations tested	Efficacy, IC_50_ (exposure time)	References
Human myeloma cell	U266 and RPMI 8226	Inhibition of STAT activation cascade, decreased DNA binding activities, reduced translocation of p-STAT3 and p-STAT5, inhibition of upstream kinases of STAT3 activation, suppression of IL-6 induced STAT3-dependent reporter gene expression. Downregulation of proteins involved in anti-apoptosis, angiogenesis, and proliferation, activates caspase-3 and cause PARP cleavage. Inhibition of cell cycle, reduced expression of cyclin D1 and cyclin B1. Induction of oxidative stress, and inhibition of glutathione reductase protein expression.	50, 75,100 µM	U266: > 100 µM (24 h)	(Kim et al., 2018a)
Human ovarian cancer cell	ES2 and OV90	Inhibition of cell proliferation, induction of cell cycle arrest, induction of apoptosis, modulation of MMP and ROS production, and regulation of ERK1/2, P38 MAPK and PI3K/AKT signal transduction.	10, 20, 40 µM	ES2: ∼40 µM (48 h) OV90: 20–40 µM (48 h)	(Park et al., 2018)
	A2780 and SKOV3	Anti-proliferation, apoptosis-inducing, depolarisation of mitochondrial membrane potential, increment of Bax/Bcl-2 ratio, suppression of metastasis, and regulation of MMP-2 and MMP-9 protein expressions and inactivation of ERK signaling.	20, 40, 80, 160, 240 µM	A2780: 310.0 µM (24h), 186.1 µM (48 h) SKOV3: 283.5 µM (24h), 209.3 µM (48 h)	(Zhang et al., 2018a)
Human colon cancer cell	HCT-116 and LoVo	Inhibition of MMP-2 and MMP-9 protein expressions.	200 µM	NA	(Auyeung et al., 2012)
	HCT-116 and HT-29	Inhibition of cell growth, apoptosis-inducing, and downregulation of NAG-1 protein expression.	6.25–400 µM, 100, 200, 400, 800 µg/ml	HCT116: 50–200 µM (24 h, 48 h, 72 h)	(Auyeung and Ko, 2010)
	SW-1116, HCT-116	Induction of cell cycle arrest, inhibition of cell growth, suppression of cell invasion, upregulation of miR-149 expression, and downregulation of EphB3, p-AKT, p-P13K, p-STAT3, inhibition of cyclin D1, MMP2/9	20, 50, 100, 200 µM	SW1116: 50–100 µM (24 h), ∼50 µM (48 h), < 50 µM (72 h) HCT116: 100–200 µM (24 h), ∼50 µM (48 h), 20–50 µM (72 h)	(Wang et al., 2018)
	RKO	Anti-proliferation, apoptosis-inducing, upregulation of Bax mRNA expression, and downregulation of Bcl-2 protein expression and p-ERK level.	20, 40, 80 µM	RKO: 20–40 µM (24 h), ∼20 µM (48 h)	(Huang et al., 2015)
Human nasopharyngeal carcinoma cell	CNE1 and CNE2	Increment of Bax and caspase-3 mRNA expression, increment of p-JNK1/2, p-p38, Bax and caspase-3 protein expressions, reduction of p-AKT and Bcl-2 protein expressions	5, 10, 20, 40 µM	CNE1 and CNE2: ∼10 µM (24 h, 48 h, 72 h)	(Qi et al., 2016)
Human breast cancer cell	ER-positive: MCF-7 and T-47D ER-negative: MDA-231, MDA-435	Anti-proliferation, apoptosis-inducing and regulation of ERβ and miR-375.	25, 50, 100 µM	MCF7: > 100 µM (24 h), ∼100 µM (48 h), 50–100 µM (72 h) T47D: > 100 µM (24 h, 48 h), 50–100 µM (72 h) MDA231: > 100 µM (24 h, 48 h, 72 h) MDA435: > 100 µM (24 h, 48 h, 72 h)	(Chen et al., 2013)
	MDA-MB-231-luc and 4T1	Inhibition of cell migration and invasion, elevation of TIMP-1 and TIMP-2, and suppression of PI3K/AKT signaling	2.5, 5, 10, 20, 40, 60, 80, 160 µM	MDA-MD-231 & 4T1: > 180 µM (24 h)	(Zhou et al., 2014)
	MCF-7, T47D, MDA-MB-435S	Anti-proliferation, apoptosis-inducing, increment of Bax, Ras, Raf, p-p38 expressions, reduction of Bcl-2 expression	25, 50, 100 µM	T47D: > 100 µM (24 h), 50–100 µM (48 h, 72 h) MCF-7: > 100 µM (24 h, 48 h), 50-100 µM (72 h) MDA-MB-435S: > 100 µM (24 h, 48 h, 72 h)	(Chen and Sun, 2012)
Human prostate cancer cell	PC-3 and DU145	Induction of cell cycle arrest, downregulation of CDK4 and cyclin D1 mRNA expressions, and reduction of CDK4, cyclin D1, and AKT protein expressions.	10, 20, 30, 40, 60, 80, 100 µM	PC-3: ∼60 µM (48 h) DU145: ∼80 µM (48 h)	(Li et al., 2014)
	LNCaP and PC-3	Anti-proliferation, apoptosis-inducing, increment of Bax mRNA and protein expression, and reduction of p-ERK1/2 protein expression.	20, 40, 80 µM	LNCaP: > 80 µM (24 h), ∼80 µM (48 h), 40–80 µM (72 h) PC-3: > 80 µM (24 h, 48 h), ∼40 µM (72 h)	(Ye et al., 2012)
	PC-3	Suppression of proliferation, apoptosis-inducing, decrement of Bcl-2 expression, increment of Bax protein expression, upregulation of p-p38 expression and downregulation of p-AKT expression	25, 50, 100 µM	PC-3: ∼25 µM (24 h, 48 h, 72 h)	(Zhang et al., 2014)
		Apoptosis-inducing, upregulation of Bax mRNA levels, inhibition of p-IGF-1R expression	25, 50, 100 µM	PC-3: 88.3 µM (48 h)	(Huang et al., 2014)
	DU145	Apoptosis-inducing, upregulation of Bax and RASD1, downregulation of Bcl-2 expression	25, 50 and 100 µM	DU145: 50–100 µM (48 h)	(Liu et al., 2014a)
Human osteosarcoma cell	U2OS	Anti-proliferation, apoptosis-inducing, inactivation of ERK and AKT, inhibition of Bcl-2 expression and increment of Bax expression, downregulation of miR-375 expression level.	5, 10, 20, 30, 40, 60, 80, 100 µM	U2OS: 60–80 µM (48 h)	(Liu et al., 2014b)
	U2OS	Anti-proliferation, downregulation of miR-375 and Ki-67 expressions, apoptosis inducing, downregulation of p-PI3KCA and p-AKT expressions.	25, 50, 100 µM	U2OS: 50–100 µM (72 h)	(Hu et al., 2019)
Human bladder cancer cell	T24	Anti-proliferation, apoptosis inducing, inhibition of cell invasion, regulation of miR-21, PTEN expressions and the phosphorylation of AKT.	50, 100, 200 µM	T24: 100–150 µM (24 h), 50–100 µM (48 h, 72 h)	(Wu et al., 2017)
Human cervical cancer cell	HeLa	Apoptosis-inducing and inhibition of PI3K/AKT signaling.	1, 5, 10, 25, 50 µM	HeLa: > 50 µM (24 h)	(Jin et al., 2014)
Human non-small cell lung cancer cell	A549 and NCI-H23	Anti-proliferation, induction of cell cycle arrest, apoptosis-inducing, downregulation of cyclin D1 and cyclin A expression levels and elevation of p53 expression	50, 100, 150, 200, 250 µM	A549 and NCI-H23: > 200 µM (12 h), 150–200 µM (24 h), 100–150 µM (48 h)	(Yang et al., 2014)
Human hepatoma cell	HuH-7	Apoptosis-inducing, increment of caspase-3 activity	20 µM	NA	(Mansoor et al., 2011)

NA, Not available

Although most studies demonstrated the anticancer effect of formononetin, there were two studies suggesting a potential cancer-promoting effect of formononetin at low concentration (<6 μM). Formononetin was reported to stimulate proliferation of nasopharyngeal CNE2 cells at 0.3 μM by activating ERK1/2 signaling pathway and upregulation of Bcl-2 anti-apoptotic protein (Guo et al., 2016). Another recent study reported that formononetin at 1–6 μM stimulated proliferation of estrogen receptor alpha (ERα)-positive breast cancer cell lines (MCF-7 and BT474) but did not affect the proliferation of ERα-negative breast cancer cell (MDA-MB-231) (Chen et al., 2018a). Considering that different doses of formononetin appear to elicit differential effects on the molecular signaling pathways, it is crucial to select the appropriate dose of formononetin in the treatment of specific cancer types. Therefore, caution should be taken when using formononetin as an adjuvant or a small molecule in chemoprevention or chemotherapy. Overall, formononetin exhibits promising anticancer potential against different cancer cells *in vitro*.

### *In Vivo* Studies

Formononetin is also able to effectively inhibit tumor growth in *in vivo* studies (Auyeung et al., 2012; Jin et al., 2014; Li et al., 2014; Hu and Xiao, 2015; Kim et al., 2018a; Hu et al., 2019). **Table 3** summarizes the *in vivo* studies on the antitumor effect of formononetin. Different types of tumors have been shown to be inhibited by formononetin, including the multiple myeloma, breast, colon, prostate, bone, and nasopharyngeal tumors. The majority of the studies employed xenograft tumor models developed by subcutaneous implantation of cancer cells into nude mice. Usually, formononetin is administered into the mice intraperitoneally or intragastrically, mainly at doses between 10 and 60 mg/kg for 2–3 weeks *via* intraperitoneal route and between 15 and 100 mg/kg for more than a month *via* intragastric route. In general, the formononetin treatment suppresses xenograft tumor growth in terms of tumor weight and volume, and also inhibits tumor invasiveness and angiogenesis. Formononetin given at a lower concentration (8 mg/kg) did not stimulate tumor growth in mice bearing MCF-7 xenografts. The study demonstrated the selective action of formononetin on the proliferation and apoptosis inhibition in vascular endothelial cells (as compared to breast cancer cells), which was associated with a feedback loop involving miR-375, RASD1, and ERα, suggesting that long-term use of formononetin is a better alternative for postmenopausal cardiovascular disease due to its lower risk for breast cancer as compared to estrogen (Chen et al., 2018a).

**Table 3:** The antitumor effect of formononetin in *in vivo* tumor bearing animal models.

Animal models	Results and mechanisms of action	Efficacy on inhibiting tumor growth/reducing tumor weight/volume	Dose and route of administration	References
Human multiple myeloma xenograft	Inhibition of tumor growth, downregulation of p-STAT3/5 expression levels, downregulation of Ki-67 expression (as a marker for inhibiting cell proliferation), inhibit angiogenesis.	Inhibition rate of tumor volume: ∼48% (20 mg/ml), ∼84% (40 mg/ml) Inhibition rate of tumor weight: ∼36% (20 mg/ml), ∼73% (40 mg/ml)	20 mg/kg, 3 times/week, i.p. 40 mg/kg, 3 times/week, i.p. Duration: 24 days	(Kim et al., 2018a)
Human colon cancer HCT-116 xenograft	Inhibition of tumor growth, inhibition of cell proliferation, decrement of invasiveness, decrement of tumor mass, reduction of VEGF expression levels in serum and tumor tissue.	Inhibition rate of tumor volume: ∼46% Inhibition rate of tumor weight: ∼36%	20 mg/kg/day, i.p. Duration: 2 weeks	(Auyeung et al., 2012)
	Reduction of tumor growth and tumor weight.	Inhibition rate of tumor volume: ∼57% Inhibition rate of tumor weight: ∼75%	15 mg/kg/day, i.g. Duration: 28 days	(Wang et al., 2018)
Human nasopharyngeal carcinoma CNE1 xenograft	Reduction of tumor volume	Inhibition rate of tumor volume: ∼40% (10 mg/kg), ∼87% (20 mg/kg) Inhibition rate of tumor weight: ∼33% (10 mg/kg), ∼78% (20 mg/kg)	10 mg/kg, every 2 days, i.p. 20 mg/kg, every 2 days, i.p. Duration: 22 days	(Qi et al., 2016)
Human prostate cancer PC-3 xenograft	Reduction of tumor growth and tumor weight.	Inhibition rate of tumor weight: 11.30% (15 mg/kg), 22.61% (30mg/kg), 45.22% (60 mg/kg)	15 mg/kg/day, i.p. 30 mg/kg/day, i.p. 60 mg/kg/day, i.p. Duration: 20 days	(Li et al., 2014)
Human breast cancer MDA-MB-231 xenograft	Reduction of tumor volume and weight, suppression of angiogenesis partly *via* FGF2/FGFR2 signaling pathway.	Inhibition rate of tumor volume: ∼67% (100 mg/kg)	100 mg/kg/day, i.g. Duration: 25 days	(Wu et al., 2015)
Human osteosarcoma U2OS xenograft	Reduction of tumor weight and growth	Inhibition rate of tumor weight: 7.75% (20 mg/kg), 30.23% (40 mg/kg), 39.53% (80 mg/kg)	20 mg/kg, i.g. 40 mg/kg, i.g. 80 mg/kg, i.g. Duration: 25 days	(Hu and Xiao, 2015)
Human osteosarcoma U2OS xenograft	Reduction of tumor mass Downregulation of miR-375 Reduced expressions of ERα, p-PI3KCA, p-AKT proteins	Inhibition rate of tumor mass: 8.03% (25 mg/kg), 32.24% (50 mg/kg), 41.56% (100 mg/kg)	25 mg/kg/day 50 mg/kg/day 100 mg/kg/day (Route of administration was not specified)	(Hu et al., 2019)
MDA-MB231-luc breast cancer xenograft	Inhibition of lung metastasis, increment of survival rate (by 30% for 10 mg/kg and 40% for 20 mg/kg).	–	10 mg/kg/day, i.p. 20 mg/kg/day, i.p.	(Zhou et al., 2014)
Human cervical tumor cell HeLa xenograft	Suppression of tumor growth, reduction of tumor weight and volume.	Inhibition rate of tumor volume: ∼17% (20 mg/kg), ∼56% (40 mg/kg) Inhibition rate of tumor weight: ∼14% (20 mg/kg), ∼34% (40 mg/kg)	20 mg/kg/day, i.g. 40 mg/kg/day, i.g. Duration: 5 weeks	(Jin et al., 2014)
Human colon carcinoma RKO xenograft	Reduction of tumor weight and volume, downregulation of TNF-α and NF-κB expressions.	Inhibition rate of tumor volume: ∼20% (5 mg/kg), ∼40% (10 mg/kg), ∼60% (20 mg/kg) Inhibition rate of tumor weight: ∼10% (5 mg/kg), ∼36% (10 mg/kg), ∼52% (20 mg/kg)	5 mg/kg/day, i.g. 10 mg/kg/day, i.g. 20 mg/kg/day, i.g. Duration: 14 days	(Huang et al., 2015)

i.p., intraperitoneal administration;

i.g., intragastric administration.

Formononetin also exhibits anti-proliferative and anti-angiogenic potential in human multiple myeloma xenograft mouse model. Administration of formononetin at 20 and 40 mg/kg three times per week *via* intraperitoneal route effectively reduced the growth of the subcutaneous model of human multiple myeloma xenograft in nude mice (Kim et al., 2018a). In addition to intraperitoneal administration, formononetin administrated *via* intragastric route at 100 mg/kg daily could suppress the growth of MDA-MB-231 breast cancer xenograft *via* angiogenesis inhibitory activity. The study demonstrated that the angiogenesis inhibitory activity of formononetin was partly associated to its modulation of FGF2/FGFR2 signaling pathway by downregulating FGF2Rα downstream molecules such as PI3K, AKT, STAT3, and MMP-2/9 (Wu et al., 2015). The promising *in vivo* anticancer properties of formononetin warrant more future research in the field of cancer chemotherapy.

## Anticancer Molecular Targets and Mechanisms of Formononetin

### Apoptosis Induction

Apoptosis is a form of programmed cell death that serves various purposes including embryonic development to maintain cell population and normal cell turnover (Elmore, 2007). Apoptosis can be triggered by either the intrinsic or extrinsic pathway. The intrinsic pathway is activated *via* internal signals. It involves Bax, Bcl-2, cytochrome C, and caspase-9. Bax protein also plays a role in inhibiting the apoptosis inhibition by Bcl-2 protein. For intrinsic apoptosis to occur, Bax protein is stimulated to move to the outer mitochondrial membrane where it forms an opening on the mitochondrial membrane; this then allows cytochrome C to migrate out of the mitochondrial intermembrane into the cytoplasm to trigger apoptosome formation, which activates caspase-9 and eventually leads to cell death (Saelens et al., 2004; Elmore, 2007). In the extrinsic pathway, apoptosis is stimulated by the external signaling pathway that involves death receptors, complement ligands, death domains, and caspase-8. A few examples of complement ligands include FasL/FasR, TNF-α/TNFR1, Apo3L/DR3, Apo2L/DR4, and Apo2L/DR5 (Chicheportiche et al., 1997; Ashkenazi and Dixit, 1998; Peter and Krammer, 1998; Suliman et al., 2001; Rubio-Moscardo et al., 2005). A simplified overview of the extrinsic pathway would be as follows: apoptotic signals are received when ligands bind to the death receptors, then death domains are activated to activate procaspase-8 into caspase-8, which will lead to apoptosis of the cell (Elmore, 2007).

There are numerous reports on the apoptosis-inducing effect of formononetin in different cancer cells. After exposure to formononetin, expression levels of cleaved caspase-3 and -9 in ovarian cancer cells increased in a dose-dependent manner (Zhang et al., 2018a). Formononetin induces apoptosis in human multiple myeloma and nasopharyngeal carcinoma cells by activating caspase-3, leading to the cleavage of poly(ADP-ribose) polymerase (PARP), which results in the inability to repair damaged DNA (Qi et al., 2016; Kim et al., 2018a). In human osteosarcoma cells and human non-small cell lung cancer, formononetin was also shown to increase the expression of caspase-3 levels in a dose-dependent manner (Liu et al., 2014b; Yang et al., 2014).

Formononetin can initiate apoptosis through a mitochondria-mediated (intrinsic) pathway in cancer cells. According to Park and his team, significant loss of mitochondrial membrane potential of approximately 457% (*P* < 0.001) and 265% (*P* < 0.001) in ES2 and OV90 ovarian cancer cells was observed upon exposure to 40 µM formononetin treatment (Park et al., 2018). Bax and Bcl-2 proteins are key regulators in intrinsic apoptosis pathway. Formononetin was reported to directly modulate the expressions of both antiapoptotic and proapoptotic members of Bcl-2 family in many cancer cells, including colon (Huang et al., 2015), nasopharyngeal (Qi et al., 2016), prostate (Ye et al., 2012; Huang et al., 2014; Liu et al., 2014a; Zhang et al., 2014), and breast cancer cells (Zhang et al., 2018a). Studies showed that formononetin induced changes in the ratio of Bax to Bcl-2 proteins in a dose-dependent manner (Hu and Xiao, 2015; Zhang et al., 2018a). The expression of Bax protein surged upon formononetin treatment, while Bcl-2 protein level decreased (Auyeung and Ko, 2010; Chen and Sun, 2012; Ye et al., 2012; Huang et al., 2014; Liu et al., 2014a; Liu et al., 2014b; Zhang et al., 2014; Huang et al., 2015). The increasing ratio of proapoptotic to antiapoptotic Bcl-2 family proteins induce the release of cytochrome *c* and other apoptogenic proteins through the mitochondrial membrane to the cytosol, subsequently leading to activation of caspase cascade and apoptosis (Heiskanen et al., 1999). Upon formononetin treatment, the percentage of A2780 ovarian cancer cells with depolarized mitochondrial membrane potential was increased with increasing concentration of formononetin (Zhang et al., 2018a). Furthermore, formononetin downregulated the expression of several other antiapoptosis mRNA and proteins such as Bcl-xL, survivin, and inhibitors of apoptosis proteins (IAP-2) (Kim et al., 2018a).

Despite the abundant evidence of formononetin-induced intrinsic apoptosis pathway, there is still no report on the apoptotic effect of formononetin mediated through the extrinsic pathways *via* stimulation of death receptors on the cell surface, such as tumor necrosis factor receptor (TNFR) superfamily. Nevertheless, a recent *in silico* study, which investigated the interactions between formononetin and death receptors, revealed that formononetin could be a potential molecule in inducing extrinsic apoptosis pathway (Vishnuvarthan et al., 2017). The study showed that formononetin displayed high affinity and steric compatibility to death receptor 5, which could be activated to mediate the TNF-related apoptosis-inducing ligand (TRAIL)-induced apoptosis (Walczak et al., 1997). This may be a focal point for future studies to investigate the possible effect of formononetin in inducing apoptosis mediated *via* the extrinsic pathways.

Oxidative stress can cause damage at the cellular level, including in the mitochondria. Increased reactive oxygen species expression creates oxidative stress that can cause opening of mitochondrial permeability transition (PT) pores and hence allow the release of cytochrome C into the cytosol to induce apoptosis (Zamzami et al., 1995; Kannan and Jain, 2000). Formononetin can also induce the apoptotic pathway *via* the overexpression of reactive oxygen species (ROS) and disturbance of the intracellular antioxidant system. The anticancer effect of formononetin on human multiple myeloma U266 cells *via* inhibition of STAT3 pathway was abrogated with the use of antioxidants such as N-acetyl-L-cysteine (NAC) and glutathione (GSH), suggesting that formononetin induces apoptosis *via* ROS production. Besides that, formononetin induced an imbalance of GSH/oxidized glutathione (GSSG) ratio through the negative regulation of glutathione reductase (GR) protein expression (Kim et al., 2018a). GR is an enzyme that catalyzes the reduction of GSSG to GSH to protect against oxidative stress (Deponte, 2013). Similarly, treatment with formononetin at 40 µM increased the production of ROS by 187% in OV90 human ovarian cancer cell (Park et al., 2018).

Nonsteroidal anti-inflammatory drug (NSAID)-activated gene (NAG-1) has anti-tumorigenic activity; hence, the overexpression of NAG-1 correlates with increased apoptotic activity (Baek et al., 2001; Eling et al., 2006). Formononetin demonstrated apoptosis-inducing effects *via* upregulating NAG-1 expression by 3- to 4-fold in human colon cancer cell HCT116 (Auyeung and Ko, 2010). The study also demonstrated that the induction of NAG-1 expression was independent of the activation of early growth response 1 (Egr-1), which is an upstream regulator of NAG-1. Taken together, formononetin is an effective apoptosis promoter in a range of cancer cells *via* several known pathways including the induction of NAG-1 and oxidative stress as well as the classical caspase-dependent pathway and modulation of Bcl-2 family of proteins.

### Cell Cycle Arrest

The eukaryotic cell cycle consists of four phases: G1, S, G2, and M phases. G1 is the phase that is vital for cell proliferation control—when the cycle proceeds from G1 phase to S phase, it is irreversible and the cell is committed to undergo division unless stresses such as DNA damage are present. Cyclin-dependent kinases (CDKs) phosphorylate a family of retinoblastoma (Rb) proteins in the G1 phase, which allows the cell cycle to proceed into S phase (Duronio and Xiong, 2013). Hence, CDKs play an important role in regulating cell proliferation. For example, cyclin-D–CDK4/6 and cyclin-E–CDK2 regulate G1/S transition while cyclin-B–CDK1 regulates transition into M phase from G2 phase (Akiyama et al., 1992; Hinds et al., 1992; Ewen et al., 1993; Kato et al., 1993). Dysregulation of the cell cycle is a main contributor to cancer, thereby resulting in uncontrolled cell proliferation. Thus, inducing cell cycle arrest represents an effective strategy to inhibit cancer.

Formononetin has been shown to induce cell cycle arrest in several types of cancer cells, such as breast (Chen et al., 2011), prostate (Li et al., 2014), lung (Yang et al., 2014), and ovarian cancer cells (Park et al., 2018). In human myeloma cells, the treatment of formononetin induced cell cycle arrest differently in U266 and RPMI 8226 cells. At 100 µM, formononetin resulted in accumulation of cells at sub-G1 phase in U266 but accumulation of cells at S phase in RPMI 8226 cells. The different phase of arrest was attributed to the differential downregulation of proteins; hence, formononetin treatment reduced expressions of cyclin D1 and cyclin B1 in U266 and RPMI 8226 cells, respectively (Kim et al., 2018a).

In ovarian cancer cells, the formononetin treatment induced significant accumulation of cells at sub-G0/G1 phase with decreased cell populations at G2/M phase in both ES2 and OV90 cells (Park et al., 2018). Similarly, formononetin also increased the percentage of PC-3 human prostate cancer cell at G0/G1 phase with downregulations of cyclin D1 and CDK4 in a dose-dependent manner (Li et al., 2014). Formononetin also induced G1 cell cycle arrest with reduced cell populations at S phase in human non-small lung cancer cells (A549 and NCI-H23) (Yang et al., 2014). Besides the downregulation of G1-phase cell cycle regulatory proteins including cyclin D1 and cyclin A, formononetin could induce the upregulation of CDK inhibitor, p21 protein expression. Clearly, majority of the studies published in this area to date demonstrate that treatment of formononetin induces sub-G0/G1 and G1 cell cycle arrest in cancer cells by modulating the expressions of cyclin regulatory proteins, including cyclin D1, cyclin E, CDK2, and CDK4 (Yang et al., 2014; Kim et al., 2018a; Park et al., 2018).

### Effect of Formononetin on Signal Transducer and Activator of Transcription (STAT) Signaling

Signal transducer and activator of transcription (STAT) proteins are responsible for modulating the expression of genes related to cell apoptosis, cell survivability, and proliferation (Battle and Frank, 2002). Once the STAT proteins are phosphorylated by either Janus kinase (JAK) or one of the Src family of protein tyrosine kinases, the activated STAT proteins dimerize and translocate to the nucleus where they bind to their target DNA to induce transcription of genes related to cell survivability. An aberrant activation of STAT proteins has been reported at a high frequency in various types of solid and liquid tumors, particularly STATS 1, 3, and 5 in acute myeloid leukemia, multiple myeloma, breast, head and neck, prostate, and lung cancer (Bromberg, 2002). Thus, STAT represents an important target for cancer prevention and treatment, whereby STAT proteins regulate the repertoire of genes associated with cancer development and progression.

One of the strategies to prevent the activation of STAT proteins, thus preventing the promotion of transcription, would be the inhibition of tyrosine kinase activity, which is itself activated by growth factor receptor, as well as members of the Src and JAK family. Upon formononetin treatment, the activation of STAT3/5 pathway was inhibited in multiple myeloma cells, as evidenced by reduced levels of expression of phosphorylated STAT3 proteins (Tyr705 and Ser727) and p-STAT5 (Tyr694/Tyr699) proteins upon formononetin treatment, but the treatment did not affect the total STAT proteins level. The study also demonstrated that formononetin mediated STAT protein inactivation *via* downregulation of several upstream signaling molecules such as JAK1, JAK2, and Src kinase (Kim et al., 2018a). Furthermore, formononetin was shown to reduce the protein expression of phosphorylated STAT3 protein in colon cancer cells, SW1116 and HCT116 (Wang et al., 2018).

Numerous reports state that IL-6 plays an essential role in the malignant progression of multiple myeloma, whereby the engagement of IL-6 with specific surface cytokine receptors activates JAK and subsequently triggers the activation of STAT3 signaling pathways (Kawano et al., 1988; Klein et al., 1995). In addition to inhibitory effects on the activation of JAK and Src kinase, the study by Kim et al. further demonstrated the inhibitory effect of formononetin on the interleukin-6 activated phosphorylation of STAT3 in multiple myeloma cells. The formononetin treatment also inhibited IL-6-induced transcription of STAT3 gene (Kim et al., 2018a).

Interfering with DNA binding activity and suppressing nuclear translocation of activated STAT homodimers will affect the transcription of regulatory genes involved in proliferation of cells. DNA binding ability of STAT3 and STAT5 was reduced in a dose- and time-dependent manner upon formononetin treatment. Moreover, formononetin also decreased the translocation activity of p-STAT3 and p-STAT5 (Kim et al., 2018a). All these findings in combination suggest that formononetin could be a promising candidate for the development of anti-cancer therapy targeting the constitutive or inducible activated STAT signaling pathway.

### Regulation of ERK1/2, P38 MAPK Signaling Pathway

Mitogen-activated protein kinase (MAPK) signaling pathway involves transduction of environmental and developmental signals that trigger cellular responses, including survival, proliferation, differentiation, inflammation, and apoptosis. A core of three-kinase cascades is involved in the activation of MAP kinases—MAP kinase kinase kinase (MAP3K) activates MAP kinase kinase (MAP2K), which subsequently activates MAP kinases. Typically, the MAPKs consist of three different groups in mammalian cells, namely, extracellular signal-regulated kinase (ERK), c-Jun N-terminal kinase (JNK), and p38 kinase.

Among the mammalian MAPK pathways, the Ras–Raf–MAPK/ERK pathway is the most well-studied and is dysregulated in various human cancers. The increased activity of ERK is frequently associated to cell proliferation and many other aspects of tumor phenotype. For instance, higher quantity of ERK1/2 was found in invasive compared to non-invasive cancer cells (Krueger et al., 2001). The constitutive activation of this pathway in cancer is caused by overexpression of receptor tyrosine kinases as well as by mutations mainly in the *RAS* and *RAF* genes (Santarpia et al., 2012). Given the significant role of ERK pathway in tumorigenesis, numerous studies have focused on exploring this pathway for development of targeted cancer treatment. In addition to clinically available compounds targeting ERK pathway, formononetin was demonstrated to be a promising molecule that inhibits the phosphorylation of ERK1/2 itself as well as the phosphorylation of downstream ERK substrate (P90RSK) (Park et al., 2018). The inhibition of ERK1/2 phosphorylation mediated by formononetin was reported in a number of cancer cells, including prostate cancer (LNCaP cells) (Ye et al., 2012), ovarian cancer (ES2, OV90 and A2780 cells) (Park et al., 2018; Zhang et al., 2018a), colon cancer (RKO cells) (Huang et al., 2015), and osteosarcoma (U2OS cells) (Liu et al., 2014b). The inactivation of ERK1/2 represents a promising anticancer strategy as activated ERK1/2 phosphorylates numerous cytoplasmic and nuclear targets, which regulate cellular processes such as proliferation, differentiation, survival, migration, and angiogenesis (Dhillon et al., 2007). Meanwhile, a contradictory finding was demonstrated in breast cancer cells in response to formononetin treatment, whereby formononetin downregulated the expression of phosphorylated ERK in MCF-7 cell (Xin et al., 2019), but no changes in p-ERK level were observed in MDA-MB-231 and 4T1 breast cancer cells (Zhou et al., 2014). This observation suggested that the inhibitory effect of formononetin on ERK pathway varies between different cancer cell types. Further data on the specific mechanism of formononetin in modulating the activation of ERK1/2 protein would be useful for future clinical development.

P38 is another important component in the 3MAPK pathway. P38 is a protein kinase that is involved in the regulation of cell differentiation, apoptosis, and autophagy (Sui et al., 2014). In contrast to the ERK pathway, the p38 pathway plays an inhibitory role in tumorigenesis and is suggested to be a tumor suppressor. Formononetin treatment increased the phosphorylation of p38 in prostate cancer (PC-3 cells) (Zhang et al., 2014), nasopharyngeal carcinoma (CNE1 cells) (Qi et al., 2016), and breast cancer (MCF-7 cells). Chen and Sun (2012) also proved that the anticancer mechanism of formononetin on breast cancer cells was linked to activation of p38 MAPK pathway. The apoptosis effect of formononetin was attenuated *via* the pretreatment of breast cancer cell with p38 inhibitor SB203580. Moreover, a study by Huang et al. (2014) suggested that activation of p38 pathway by formononetin could be mediated *via* the suppression of IGF1-R expression, subsequently leading to the activation of pro-apoptosis cascade in PC-3 prostate cancer cells. Overall, the currently available pre-clinical results indicate that formononetin modulates the MAPK pathway by inhibiting the phosphorylation of ERK1/2 as well as activating p38, subsequently contributing to the attenuation of cell proliferation and induction of apoptosis.

### Regulation of PI3K/AKT Signaling Pathway

The phosphatidylinositol 3-kinase/protein kinase-B (PI3K/AKT) signaling pathway plays an important role in regulating cell proliferation, cell survival and apoptosis, differentiation, and cellular metabolism (Liu et al., 2009). As a major survival pathway in cancer cells, the constitutively activated PI3K–AKT signaling pathway mediated through molecular aberrations drives the process of tumor promotion and resistance to chemotherapy (Liu et al., 2009; Janku et al., 2012). As a common oncogenic driver in various cancer cells, targeting this signaling pathway has been considered as one of the most attractive targets for the development of anticancer agents (Polivka and Janku, 2014; Porta et al., 2014).

The activation of AKT, a serine/threonine kinase, is induced *via* phosphorylation mediated by PI3K lipid kinase. The activated AKT regulates downstream targets, which leads to increased cell proliferation, resistance to apoptosis, metastasis, and angiogenesis (Porta et al., 2014). Thus, inhibition of PI3K or AKT represents a promising strategy, particularly against cancer cells with increased PI3K/AKT activity. Previous studies have demonstrated that formononetin exerts an inhibitory effect on PI3K/AKT signaling pathway. In the case of MDA-MB-231 and 4T1 breast cancer cells, both phosphorylated PI3K and p-AKT were downregulated upon formononetin treatment (Zhou et al., 2014). In addition, formononetin treatment downregulated p-AKT in several other types of cancer cells, including U266 multiple myeloma (Wu et al., 2016), Hela cervical cancer (Jin et al., 2014), PC-3 prostate cancer (Zhang et al., 2014), and U2OS osteosarcoma cells (Hu and Xiao, 2015). In addition, a recent *in vivo* study reported that formononetin downregulated p-PI3KCA^Tyr317^ and p-AKT^Ser473^ proteins in the U2OS osteosarcoma xenograft mice (Hu et al., 2019). Treatment with formononetin also resulted in the downregulation of downstream molecules of AKT, P70S6K, and S6 proteins in ovarian cancer (ES2 and OV90) cells (Park et al., 2018). Formononetin has also been shown to inhibit the upstream signals of the PI3K–AKT pathway by downregulating the expression of the insulin-like growth factor receptor (IGF-1R) in human breast cancer cells (Chen et al., 2011; Chen et al., 2013).

Phosphatase and tensin (PTEN) is a lipid and protein phosphatase whose major substrate is phosphatidylinositol-3,4,5-triphosphate (PIP3), a secondary messenger produced upon PI3K activation, thus working as a negative regulator of PI3K–AKT pathway (Carnero et al., 2008). Thus, PTEN acts as a tumor suppressor that functions to inhibit cell proliferation and cell invasiveness (Milella et al., 2015). The loss of function in PTEN has been associated with many common cancers (Hollander et al., 2011). Besides the inhibition on AKT activity, formononetin upregulated PTEN expression in bladder cancer T24 cell. Furthermore, formononetin induced downregulation of microRNA-21 (miR-21), which functions as an oncogene in bladder cancer, which suggests that formononetin could be an effective agent that modulates oncogenic microRNA targeting PTEN (Wu et al., 2017).

Another mechanism underlying PI3K/AKT contribution to tumorigenesis is hypoxia, whereby a hypoxic tumor microenvironment can increase AKT activity, thus conferring resistance to apoptosis. Previous literature indicated that hypoxia could induce the AKT signaling cascade, and hypoxia-inducible factor-α (HIF-1α) protein accumulation is closely associated with active AKT pathway in various hypoxic cancer cells (Ardyanto et al., 2006; Wang et al., 2014). Formononetin also mediates anticancer effect by inhibiting the phosphorylation of AKT in hypoxic multiple myeloma cell. The inhibition of AKT pathway by formononetin was mediated through the attenuation of HIF-α expression and inflammatory cytokines release, suggesting that formononetin could be a potential therapeutic agent that can prevent hypoxia-induced tumorigenesis *via* suppression of AKT pathway (Wu et al., 2016). Overall, these evidences demonstrated that formononetin serves as a promising candidate targeting different components associated with PI3K/AKT signaling pathway, which has contributed to the formation of a majority of human malignancies.

### Modulation microRNA Expression

MicroRNAs (miRNA) are small non-coding RNAs that consist of approximately 22 nucleotides and play a role in regulating gene expression, usually by gene silencing, particularly genes involved in regulation of major cellular processes (Esquela-Kerscher and Slack, 2006; He et al., 2017). MicroRNAs are well recognized for their roles in pathogenesis of human diseases, such as carcinogenesis, where dysregulated expression of miRNAs has been found in a variety of human cancers. Thus, targeting miRNA expression in cancer represents a promising anticancer strategy. For instance, upregulation of miR-375 expression is associated to tumor suppressing effect, while suppressing the expression of miR-21 which has been identified as an oncogenic microRNA and is known to be upregulated in most human cancer types (Xu et al., 2015; Wang et al., 2016; Chen et al., 2018b). One study demonstrated that both miR-375 and miR-21 in combination can be used as a prognostic biomarker better than either alone, where high miR-375 and low miR-21 expressions give a higher survivability rate (He et al., 2017).

A number of studies demonstrated that formononetin exerts anticancer effects by regulating the expression of miRNAs in cancer cells (Chen et al., 2013; Hu and Xiao, 2015; Guo et al., 2017b; Chen et al., 2018a). The inhibitory effect of formononetin on the proliferation of bladder cancer T24 cells was mediated *via* downregulation of miR-21 expression, upregulation of PTEN, and inactivation of AKT phosphorylation (Wu et al., 2017). miR-21 was previously shown to regulate PTEN expression in human hepatocellular cancer (Meng et al., 2007). A recent study also revealed a modulatory effect of formononetin on the expression of miR-149 and its direct gene target, EphB3, which then played a role in formononetin-induced inhibition of cell proliferation and invasion in SW1116 and HCT116 colon cancer cells (Wang et al., 2018).

Generally, miR-375 is known for its tumor suppressor effect, and is frequently downregulated in various cancers such as hepatocellular carcinoma, esophageal carcinoma, gastric cancer, melanoma, and glioma (Yan et al., 2014). However, there are contradictory results observed in other cancers, including breast, bone, and prostate cancers, suggesting that miR-375 functions vary in different cell types. The expression of miR-375 in osteosarcoma cell was downregulated upon formononetin treatment (Hu and Xiao, 2015; Hu et al., 2019). The inhibitory effect of formononetin on estrogen receptor (ER)-positive U2OS osteosarcoma cell was suggested to be mediated through the downregulation of miR-375, which has been associated with estrogen receptor signaling (Hu and Xiao, 2015). This finding is in line with a recent *in vivo* study reporting similar formononetin-mediated mechanism in the inhibition of U2OS osteosarcoma xenograft in mice (Hu et al., 2019). Another similar result was also observed in breast cancer cells by Chen et al. (2013), showing that formononetin (≥50 μM) decreased expression of miR-375 but was followed by enhanced estrogen receptor beta (ERβ) expression level, which has been associated to anti-proliferative effect, especially ERβ1 (Akiyama et al., 1992; Dey et al., 2012). These studies not only strengthen the role of ERα in malignancies but also suggest that regulating the levels of ERα and ERβ could be an alternative strategy in managing breast cancer.

Previous evidence also supports these findings as miR-375 expression was upregulated in ERα-positive breast cancer tissue (Simonini et al., 2010). Formononetin at lower concentrations (6 μM) could upregulate the expression of miR-375 in HUVECs but not in MCF-7 or BT474 breast cancer cells. This result reflects the weaker effect of formononetin on cell proliferation and anti-apoptosis in breast cancer cells as compared to HUVECs, suggesting that long-term use of formononetin could be used by postmenopausal women to alleviate symptoms of estrogen deficiency while conferring a lower risk of postmenopausal breast cancer as compared to estrogen (Chen et al., 2018a). In contrast, however, a low dose of formononetin (0.1 and 0.3 μM) was found to accelerate the proliferation of nasopharyngeal carcinoma by upregulating miR-375 expression and leading to the upregulation of ERα as well as downregulation of PTEN (Guo et al., 2016). These studies indicate that treatment with formononetin at low concentrations results in complex, cell type-specific effects in ER-positive cancer cells *via* the modulation of miRNA. These results suggest that special precautions should be considered if formononetin is used for treatment of ER-positive cancers.

### Suppression of Migration, Invasion, and Angiogenesis of Cancer Cells

Metastasis is a multistep process resulting in the spread of cancer cells to distant regions of the body *via* blood or lymphatic system; these steps include detachment, migration, and invasion. Controlling tumor cell metastasis is of critical importance in management of cancer disease, particularly in cancers detected early. Several studies have shown that formononetin can suppress metastasis of various cancer cells including colon cancer (Auyeung et al., 2012; Wang et al., 2018), bladder cancer (Wu et al., 2017), ovarian cancer, and breast cancer (Zhou et al., 2014). For example, the migratory ability of ovarian cancer cells was reduced by 30.41% and 57.34% after treatment with 20 and 40 µM of formononetin, respectively. The work also demonstrated inhibition of invasiveness of ovarian cancer cells by 41.85% and 73.75% after exposure to formononetin at 20 and 40 µM, respectively (Zhang et al., 2018a). In addition, formononetin also inhibited the invasive and migratory properties of breast cancer cells MDA-MB-231 and 4T1 (Zhou et al., 2014).

Several studies have investigated the underlying mechanism of formononetin in suppressing metastasis of cancer cells. Formononetin also demonstrates an inhibitory effect on the expression of matrix metalloproteinases (MMPs) such as MMP-2 and MMP-9 proteins, which play an essential role in the metastatic process of tumor cells as well as the regulation of angiogenesis in the maintenance of tumor cell survivability (Ly et al., 2003; Auyeung et al., 2012; Zhou et al., 2014; Wang et al., 2018; Zhang et al., 2018a). MMPs are a group extracellular matrix degrading enzymes that regulate numerous normal cellular processes such as cell growth, differentiation, apoptosis, and migration. However, MMP activity is elevated in many tumor cells. The overexpression of MMP-2 and MMP-9 is associated with pro-oncogenic events such as neo-angiogenesis, tumor cell proliferation, and metastasis (Alizadeh et al., 2014). Formononetin was also shown to exhibit an anti-invasive effect by enhancing the expression of the negative regulators of MMPs, such as tissue inhibitors of metalloproteinases (TIMPs) TIMP-1 and TIMP-2 in breast cancer cells (Zhou et al., 2014). Besides the modulating effects demonstrated by formononetin on the MMP and TIMP expression in several tumor cells, a recent study revealed another potential type of anti-metastasis mechanism of formononetin on cancer cells. The study showed that formononetin exhibits a high binding constant and high affinity (Δ*G* = −38.07 kJ/mol) to actin molecule. Formononetin interacts with the ATP binding site of actin molecules, suggesting that the interaction limits the conformational change and polymerization of actin molecules and hence filament formation. Therefore, formononetin could serve as a ligand that disrupts the organization of actin filaments, preventing the movement of cancer cells and therefore preventing invasion (Budryn et al., 2018).

Angiogenesis is the formation of new blood vessels for supplying nutrients and oxygen to tissues and cells. In tumorigenesis, angiogenesis is important for the development and progression of malignant tumors. Vascular endothelial growth factor (VEGF) and fibroblast growth factor-2 FGF2 are among the factors that play an important role in tumor angiogenesis. The formononetin treatment downregulated gene and protein expressions of VEGF in HCT116 colon cancer cells (Auyeung et al., 2012). Formononetin also showed great inhibitory effect on fibroblast growth factor receptor 2 (FGFR2) with 89% reduction noted at 1 µM. Using HUVEC as an *in vitro* model, formononetin reduced the stimulatory activity of FGF2 on FGFR2, resulting in downregulation of signaling pathways of FGFR2 such as phosphorylation of AKT and PI3K. Invasiveness and proliferation of HUVEC in response to FGFR2 activation were thus potentially inhibited by formononetin. However, FGFR1 and its downstream activities were not affected by formononetin, suggesting that formononetin suppresses angiogenesis *via* the FGFR2 signaling pathway. In line to the *in vitro* analysis, formononetin also inhibited tumor angiogenesis *in vivo* in a human breast cancer xenograft mouse model. The result supported the *in vitro* findings where formononetin suppressed angiogenesis *via* inhibition of microvessel density and reduction of phosphorylated FGFR2-positive cells in the tumors (Wu et al., 2015). Overall, with these characteristics, more studies should be conducted to validate formononetin’s efficacy in suppressing tumor invasion, metastasis, and angiogenesis.

## Combinatorial Use of Formononetin With Other Chemotherapeutic Drugs

Multidrug resistance is currently not an uncommon occurrence hindering the efficacy of clinical anticancer drugs. As a result, combinatorial treatment has been given significant attention in cancer therapy looking into how a combination of anticancer agents could work additively or synergistically to confer enhanced antitumor activities at lower doses compared to single drug treatment (Baxevanis et al., 2009; Oak et al., 2012; Arshad and Datta, 2017; Wagenaar et al., 2018). The rationale behind the combination chemotherapy is to co-administer drugs that function by different molecular mechanisms, thus enhancing tumor suppression while reducing the likelihood of drug resistance and side effects (Al-Lazikani et al., 2012). Given the promising anticancer properties demonstrated by formononetin, there have been numerous studies conducted to evaluate its potential to be used in combination to confer synergistic effects with other chemotherapeutic drugs, including bortezomib, LY294002, U0126, sunitinib, epirubicin, doxorubicin, temozolomide, and metformin.

Formononetin was shown to potentiate bortezomib-induced apoptosis in multiple myeloma (Kim et al., 2018a). Bortezomib is the first proteasome inhibitor approved for treating hematologic malignancies by inhibiting the proteolytic function of proteasome; in combination with formononetin, the inhibition of STAT3 induced by bortezomib was further enhanced, resulting in a 3-fold increase of apoptosis in U266 cells. Synergistic effects were also observed when formononetin was used in combination with pharmacological inhibitors like LY294002 (PI3K inhibitor) and U0126 (ERK1/2 inhibitor). The combination of formononetin with both LY294002 and U0126 demonstrated enhanced inhibitory effects on the proliferation of ovarian cancer ES2 and OV90 cells (Park et al., 2018). Recently, synergistic effect was evident between formononetin and metformin in cancer treatment. Metformin, which is well known for treatment of type 2 diabetes, has recently emerged as a potential anticancer agent. However, metformin could induce side effects in non-diabetic patients at high concentrations (5–30 mM). Xin et al. (2019) demonstrated that the antiproliferative effect of formononetin was enhanced when used in combination with metformin in MCF-7 breast cancer cell. The synergistic effect demonstrated by the combination of formononetin and metformin was attributed to the downregulation of the ERK1/2 signaling pathway.

Anthracyclines represent a class of powerful antitumor agents used for the treatment of solid tumors, leukaemia, and lymphoma. However, the use of anthracyclines is limited by associated cardiotoxic effects and development of drug resistance. There were several studies evaluating the cytotoxicity enhancing effect of formononetin towards anthracyclines in chemotherapeutic applications. Formononetin potentiated the cytotoxic efficacy of epirubicin against cervical cancer HeLa cells (Lo and Wang, 2013) and breast cancer MDA-MB-231 cells (Gyémánt et al., 2005). The studies demonstrated that the administration of formononetin inhibited the efflux transporter-mediated epirubicin resistance by modulating the gene expression of multi-drug receptor (MDR) transporters [MDR1, MDR-associated protein (MRP)1, and MRP2] (Lo and Wang, 2013) as well as inhibiting P-glycoprotein efflux pump-mediated resistance (Gyémánt et al., 2005).

On the other hand, the sensitivity of glioma cells towards doxorubicin was shown to be enhanced by co-treatment with formononetin. The doxorubicin-induced epithelial-mesenchymal transition (EMT) in glioma cells can be reversed by formononetin as evidenced by the changes of EMT markers such as decreased vimentin and increased E-cadherin levels. The reversal of EMT induced by formononetin in doxorubicin-treated glioma cells was associated with formononetin’s suppressive effect on the histone deacetylase (HDAC) 5 expression, subsequently leading to reduced proliferation of the glioma cells (Liu et al., 2015). In addition to that, formononetin also worked synergistically with temozolomide against glioma C6 cells. The combination of formononetin with temozolomide enhanced apoptosis and inhibited migration of glioma cells (Zhang et al., 2018b).

Besides the promising combinatorial effects demonstrated between formononetin and other anticancer agents in *in vitro* models, Wu et al. (2015) reported a more significant effect of combination treatment of formononetin and sunitinib (VEGFR2 inhibitor) in inhibiting the tumor growth in a mouse xenograft breast cancer model as compared to treatment with formononetin or sunitinib alone. Therefore, more preclinical studies on different combinations are warranted to ascertain the usefulness of formononetin as an adjuvant in chemotherapeutic applications.

## Metabolism, Bioavailability, and Pharmacological Relevance of Formononetin

Due to its lipophilic nature, formononetin is rapidly absorbed into the gut *via* passive diffusion, with a peak absorption at 30 min (Luo et al., 2018). Parallel artificial membrane permeability assay (PAMPA) showed that formononetin exhibited high permeability at pH 4.0 and 7.0 (Singh et al., 2011). Small intestine was the main absorption site of formononetin before reaching the large intestine (Luo et al., 2018). During first pass metabolism, formononetin is rapidly *O*-demethylated into daidzein before being rapidly conjugated in phase II metabolism. The plasma concentration time curve (AUC) of formononetin conjugates (glucuronides and/or sulfates) was higher than free formononetin at a given time post-administration, indicating that its conjugate form is the dominant form after oral and intravenous administration (Singh et al., 2011).

Several studies reported the pharmacokinetics and bioavailability of formononetin after different routes of administration in a rat model (Singh et al., 2011; Luo et al., 2018). Formononetin was determined to have a half-life of ∼2–3 h after oral administration and ∼2 h after intravenous administration. At oral administration of formononetin at 20–50 mg/kg, the peak plasma concentration was achieved between (T_max_) 0.5–1 h, while the maximum plasma concentration (C_max_) was determined ranging from 62 nM (17 ng/ml) to 302 nM (81 ng/ml). Meanwhile, the C_max_ of 1,302.8 nM (349.5 ng/ml) and 16,956.6 nM (4,548.5 ng/ml) was achieved after intravenous administration of formononetin at 4 and 10 mg/kg, respectively (Singh et al., 2011; Luo et al., 2018). The clearance of formononetin was reported to be ∼400 L/h/kg for oral administration and ∼5 L/h/kg for intravenous administration. Upon oral administration at 20 mg/kg, formononetin has a bioavailability of ∼22% (Singh et al., 2011; Li et al., 2016b; Luo et al., 2018). These data showed that formononetin, no difference from other isoflavones, is rapidly metabolized and extensively converted into its metabolites daidzein and conjugates of daidzein and formononetin to be excreted, which makes it poorly bioavailable.

The above information may be applied to help design *in vitro* studies that investigate formononetin at clinically realistic and physiologically achievable doses. Based on the literature, the concentration required to elicit an anticancer response in the majority of *in vitro* studies is considerably higher than the levels that could be realistically achieved *in vivo*. This then raises the question as to the actual potential usefulness of formononetin as an anticancer agent in an actual clinical setting, but given that formononetin has clearly demonstrated antitumor efficacy in several human tumor xenograft animal models, it does appear to be a molecule worthy of further investigation.

Biotransformation, which plays a critical role in the pharmacological activities of orally administered compounds, may be partially responsible for the higher than predicted *in vivo* efficacy of formononetin. Enterohepatic recycling of phase II conjugates may increase its anticancer properties at a particular dose as it may result in increased time of exposure of formononetin to the target cells. It has previously been postulated that the antitumor effect of formononetin could be attributed to the prolonged contact time between formononetin and target tissues (Zeng et al., 2016). Enterohepatic recycling refers to the process of re-entry of conjugates into the intestinal tract *via* biliary excretion, which is a continuous cycle as the microflora enzymes catalyze these conjugates back to aglycones for reabsorption.

Also, in contrast to single exposure *in vitro* studies, daily administration of formononetin was performed in most of the *in vivo* studies, and long-term exposure to dietary flavonoids may produce significant concentrations in plasma and tissues even if the intake levels are low (D’archivio et al., 2010). In addition, synergistic effects could exist between formononetin and its metabolites formed *in vivo*, an additional area that may warrant further investigation. Given these factors, the maximum plasma concentration of formononetin alone may not be an absolute determinant for estimating anticancer effects of formononetin *in vivo*.

## Potential Drug Delivery of Formononetin

Drug delivery systems are strategies employed to overcome the low bioavailability and low water solubility of formononetin to achieve its pharmacological efficacy at minimum dose. A number of efforts have had promising results in developing efficient drug carriers to deliver formononetin to the target site, including the hydroxypropyl-β-cyclodextrin-modified carboxylated single-walled carbon nanotubes (CD-SWCNTs) (Liu et al., 2018), multiwalled carbon nanotube (Guo et al., 2018), poly(lactic-*co*-glycolic acid) (PLGA)-nanoparticle loaded with formononetin hydroxypropyl-β-cyclodextrin complex (Guo et al., 2017a), and _D_-α-tocopheryl polyethylene glycol 1000 succinate (TPGS) micelles containing formononetin (Cheng et al., 2016). These drug delivery methods have successfully improved the solubility and absorption of formononetin.

The CD-SWCNTs were synthesized by grafting hydroxypropyl cyclodextrin to carboxylated single-walled carbon nanotubes. This mode of delivery increased the biocompatibility and reduced the toxicity of carbon nanotubes. The use of CD-SWCNTs as the carrier of formononetin enhanced its cytotoxic ability toward breast cancer MCF-7 and cervical cancer HeLa cells as compared to free formononetin. Furthermore, this intervention also provided improvement in homogenous dispersibility and sustained-release properties (Liu et al., 2018). Within the year 2018, Guo et al. (2018) reported the development of a delivery system for entrapment of formononetin–multiwalled carbon nanotube–formononetin (MWCNT-FMN) conjugates exhibiting apoptosis-inducing effect in HeLa cervical cancer cells.

Yet another intervention was developed by incorporating formononetin in 2-hydroxypropyl-β-cyclodextrin inclusion complex loaded in PLGA-nanoparticles with a size of ∼200 nm. The intervention was able to exert a sustained release effect with cumulative release of 50% of formononetin over 24 h. However, the cytotoxicity of this intervention was slightly weaker as compared to free formononetin, and this was suggested to be due to incomplete release of formononetin from the intervention (Guo et al., 2017a).

Another method of drug delivery was the incorporation of formononetin into phospholipid/vitamin E TPGS micelles with high tumor targeting efficiency. This drug delivery system was shown to enhance the drug cellular uptake and cell cytotoxicity in *in vivo* xenograft lung tumor model in mice. Micelles with size of ∼100 nm together with hydrophilic surface modification allowed the drug to evade the phagocytic system, hence achieving sustained released properties with cumulative release of 45% of formononetin over 120 h. Despite a lower antitumor efficacy of formononetin micelles as compared to cisplatin, this intervention was demonstrated to be much safer in the *in vivo* antitumor experiment with no weight loss and high survival rate (80% after 14 days) of the mice (Cheng et al., 2016).

## Future Perspective

Currently, numerous preclinical investigations have reported and validated independently that formononetin exhibits chemopreventive and therapeutic potentials against a wide range of cancers. However, there is still insufficient evidence to delineate the exact anticancer mechanisms of formononetin and to facilitate its clinical application in the treatment of human cancer. Thus, future studies should concentrate on decoding the precise anticancer mechanisms of formononetin. During the past few years, the surge of the “omics” technologies has enabled the identification and elucidation of biological changes in response to perturbations in cells and tissues (Turanli et al., 2018). In addition to conventional *in vitro* assays, more global and powerful approaches such as proteomics, transcriptomics, or metabolomics are required to provide comprehensive insight into integral perturbed biomolecule profiles of cancer cells in response to formononetin treatment.

Given that formononetin undergoes extensive metabolism in the body, it is crucial to identify the circulating metabolites correctly to have a better understanding of the fate of formononetin upon consumption. This would help build a more complete picture of the overall bioactivity of formononetin and allow correlation between the bioactivity of the parent molecule and its circulating metabolites with the target tissue. In order to improve our understanding of the potential cellular mechanisms of action of formononetin, it is strongly recommended to improve the design on the future *in vitro* studies to mimic more achievable *in vivo* conditions by taking into account the actual metabolites and concentrations detected in the respective tissues. It is also essential to identify whether ingested formononetin reaches the target tissues. However, up to now, only a few studies have attempted to determine formononetin and its metabolites qualitatively and quantitatively in humans or even in experimental animal tissues. Moreover, conjugates of formononetin are the major circulating flavonoids rather than the glycosides or the aglycones that have been extensively studied *in vitro* (Singh et al., 2011). Unfortunately, the pharmacological roles of these conjugates are not well understood.

Gut microbiota play a significant role in biotransformation and degradation of isoflavones in humans (Landete et al., 2016). Given that the composition of gut microbiota differs considerably between individuals, the highly variable process of biotransformation mediated by gut microbiota in humans could have a substantial impact on plasma concentrations of formononetin and its metabolites, subsequently leading to differential biological effects. Thus, the impact of gut microbiota on the bioavailability of formononetin and its metabolites should also be taken into consideration for further pharmacological use. More research should be performed to verify the anticancer properties of formononetin, with special attention given to the minimum effective dose and its toxicity, in order to provide deeper understanding of the role of formononetin in chemoprevention and chemotherapy.

## Conclusion

**Figure 2:**
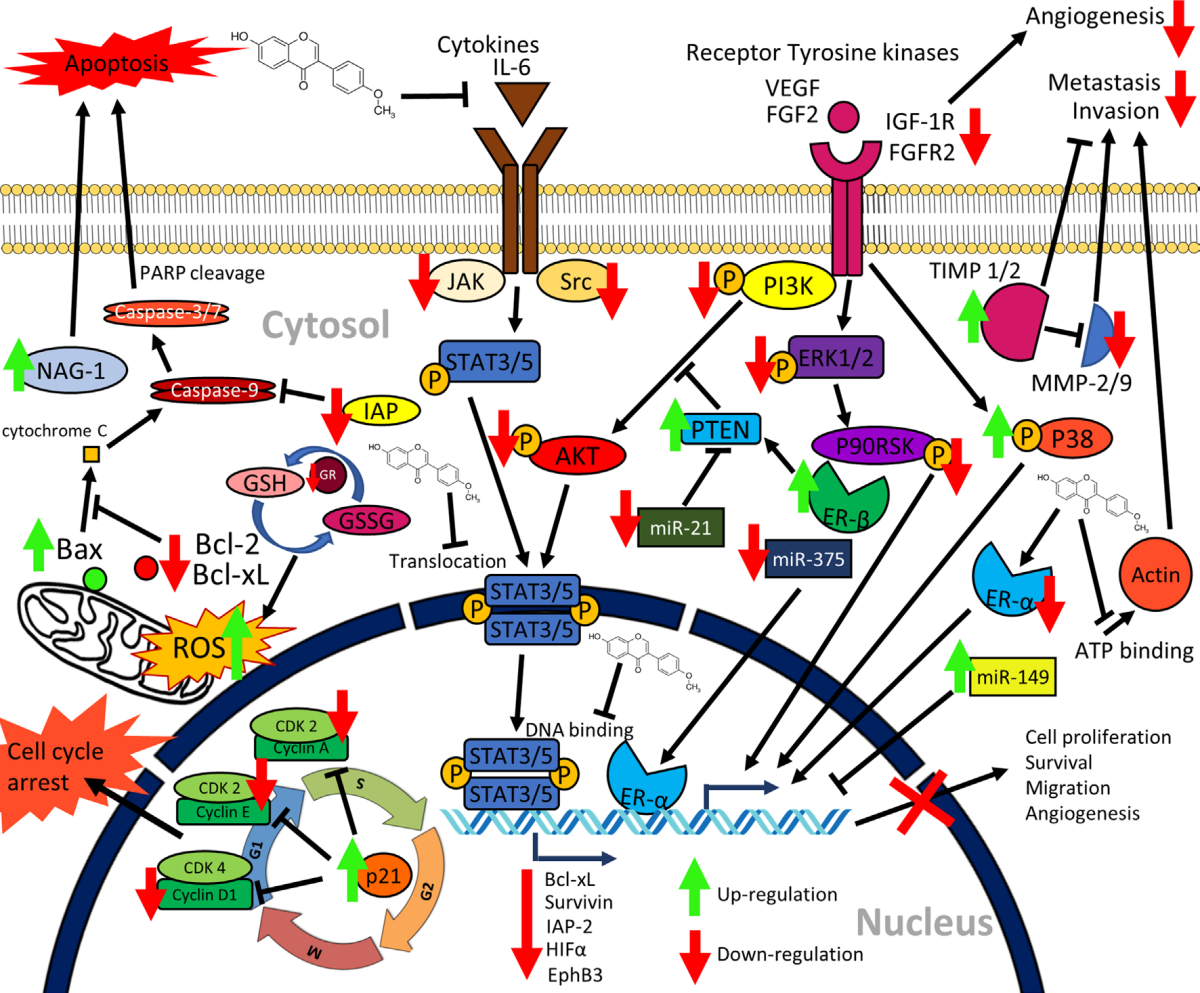
Graphical summary of the anticancer mechanisms of formononetin. Formononetin acts on multiple signaling pathways in cancer cells. It induces apoptosis *via* classical caspase-dependent pathway and modulation of Bcl-2 family of proteins. It induces cell cycle arrest by modulating the cycle regulatory proteins. It inactivates signaling pathways, namely, JAK/STAT pathway, PI3K/AKT pathway, as well as MAPK (ERK1/2) pathways. Formononetin also modulates several miRNA expressions as well as supresses cell migration, invasion, and angiogenesis (Li et al., 2014; Liu et al., 2014b; Yang et al., 2014; Hu and Xiao, 2015; Guo et al., 2016; Qi et al., 2016; Wu et al., 2017; Kim et al., 2018a; Park et al., 2018; Wang et al., 2018; Zhang et al., 2018a). NAG-1, nonsteroidal anti-inflammatory drug (NSAID)-activated gene-1; IAP, inhibitors of apoptosis proteins; IL-6, interleukin-6; PARP, poly(ADP-ribose) polymerase; GSH, glutathione; GSSG, oxidized glutathione; GR, glutathione reductase; PTEN, phosphatase and tensin; HIFα, hypoxia inducible factor α; miR, microRNA; TIMP, tissue inhibitors of metalloproteinases; ER, estrogen receptor; MMP, matrix metalloproteinases; CDK, cyclin dependent kinases; STAT, signal transducer and activator of transcription; PI3K, phosphatidylinositol 3-kinase; AKT, protein kinase B; VEGF, vascular endothelial growth factor; FGF, fibroblast growth factor; FGFR, fibroblast growth factor receptor; IGF-1R, insulin-like growth factor 1 receptor; ERK, extracellular signal-regulated kinase; JAK, Janus kinase; ROS, reactive oxygen species.

A cure for cancer has remained elusive for centuries, although there are several methods to slow down or curb the progression of this disease such as surgery, chemotherapy, hormonal therapy, radiation therapy, and immunotherapy. In recent years, novel plant-based compounds have gathered significant interest with regard to their anticancer properties. We have summarized the available evidence on the promising role of formononetin against cancer. As mentioned, formononetin is one of the potential anticancer compounds that exert pleotropic effects and targeting multiple cellular processes of cancer cells. The *in vitro* studies based on different human cancer cell lines confer deeper insights in novel molecular and cellular mechanisms of formononetin, which impede the progress of carcinogenesis and metastasis. The notable mechanisms include regulation of transcription factors, modulation of epigenetic targets, regulation of estrogen receptors, regulation of cell cycle, induction of apoptosis, and regulation of growth and developmental signaling pathways (**Figure 2**). *In vivo* studies are also promising as the majority of the *in vivo* studies of formononetin are based on human cancer xenografts, including myeloma, colon, and prostate cancers. These *in vivo* findings support the anticancer potential of formononetin by inhibiting tumor growth and inducing tumor cell apoptosis. Considerable attention has also been given to improve formononetin properties with the developments of various drug delivery systems. This review concludes that formononetin may be considered as a potential candidate for anticancer drug discovery or dietary supplements and nutraceuticals.

## Author Contributions

The writing was performed by K-CT, LT-HT, WHY, PP, CKC, SLH, L-HL, and B-HG. B-HG and LT-HT provided vital guidance and insight to the work. KG-C, WHY, L-HL and B-HG contributed to the funding of the project. The project was conceptualized by B-HG..

## Funding

This work was inspired by Monash Pharmacy Degree Course, Unit PAC3512, entitled “Current aspects of pharmaceutical research” and financially supported by Taylor's University Emerging Grant (TRGS/ERFS/2/2018/SBS/016), University of Malaya Research Grants (PPP grants PG136-2016A and PG135-2016A and JBK grant GA002-2016), and External Industry Grants from Biotek Abadi Sdn Bhd (vote no. GBA-81811A).).

## Conflict of Interest Statement

The authors declare that the research was conducted in the absence of any commercial or financial relationships that could be construed as a potential conflict of interest.
